# Genomic selection strategies and their potential to maintain rare alleles and de-novo mutations: a long-term assessment

**DOI:** 10.1186/s12711-026-01052-x

**Published:** 2026-06-11

**Authors:** Matias F. Schrauf, Jeremie Vandenplas, Herman A. Mulder, Yvonne C. J. Wientjes

**Affiliations:** Animal Breeding and Genomics, Wageningen University and Research, Wageningen, The Netherlands

## Abstract

****Background**:**

Sustainable breeding programs need to balance short-term genetic improvement with the conservation of genetic diversity. While genomic selection has considerably increased the genetic gain for many breeding programs, the consequences on diversity can be less desirable. This is particularly the case for rare alleles and de-novo mutations, as markers used in genomic selection are generally not strongly associated with rare alleles. Moreover, genomic selection allows for the selection of young individuals without records, thereby ignoring the effects of de-novo mutations. We aimed to evaluate various selection strategies in terms of long-term genetic gain and conservation of genetic variance, with a focus on the use and conservation of favorable rare alleles and de-novo mutations.

****Results**:**

To study these selection strategies, we simulated populations of 1000 individuals subject to 50 generations of selection with a trait with only additive gene actions, a trait with additive and dominance gene actions, and a trait with additive, dominance and epistatic gene actions. For each trait, we evaluated five genomic selection strategies that balance between genetic improvement and diversity management, namely: truncation selection, which only focuses on short-term genetic gain; optimal contribution selection, which balances short-term genetic gain with a constraint on the relatedness of the selected individuals; two versions of allele-reweighted selection, which upscale the effect of rare alleles in the breeding values; and constrained allele loss selection, a novel strategy which balances short-term gain with a constraint on the reduction in frequency of rare alleles estimated to be favorable. Our results show that the allele-reweighted strategies provided an efficient trade-off between conserving genetic variance and achieving a higher genetic long-term gain, improving one or both metrics relative to truncation selection. Optimal contribution selection also improved the amount of genetic variance conserved and, for the trait with epistatic gene actions, also resulted in higher long-term genetic gain. On the other hand, the constrained allele loss did not show improvements over truncation selection.

****Conclusions**:**

Introducing into our genomic selection strategies a consideration for diversity management or the conservation of rare alleles can help in improving the long-term sustainability of breeding programs that use genomic selection.

## Background

Whether inherited from previous generations or generated by de-novo mutations, the appropriate use of rare genetic alleles is a challenge to the long-term effectiveness of genomic selection in breeding programs. Individually, these rare alleles only explain a small proportion of the genetic variance and their effects are difficult to estimate accurately. Thus, it is common that they are mostly ignored by selection methods and subject to be lost by random drift [1].

At the same time, these rare loci hold the highest potential change in allelic frequencies (in the direction that goes from rarity towards fixation for the rare allele). If favorable, an increase in frequency of these alleles can generate an increase in the average genetic merit of the selected populations. The risk of losing favorable alleles is more pronounced when selection candidates do not have their own phenotypic records to assist in the estimation of linked marker effects, in particular for de-novo mutations [2]. This situation is common for many traits of interest in livestock; such as for traits which are difficult to measure, only observed in one of the sexes, late in life, or both. Additionally, the use of genomic selection has also increased the occurrence of younger selection candidates with fewer records [3].

Thus, it is of interest to explore the effectiveness of different genomic selection strategies at maintaining the potentially favorable rare alleles segregating in the population. This would increase the chance for individuals carrying these alleles to gather phenotypic records; so that their allelic effects can be estimated and they can contribute to future genetic gain. Such genomic selection strategies range from aiming to reduce drift, as it can be the case with optimal contribution selection theory [4], to emphazising these rare alleles by up-scaling their estimated effects in the genomic predictions [5].

Any selection strategy in a breeding program must face a trade-off between short and long-term genetic gain. If selection strategies overly prioritize immediate genetic gain, this can result in an imporant loss of genetic diversity and limit further response to selection in the long-term. Non-additive gene actions, such as dominance and epistasis, further complicate this balance: non-additive genetic variance cannot be directly harnessed by selection under random mating, but it can be converted to additive variance through changes in allele frequencies across generations, potentially adding to long-term genetic gain [6, 7].

In the present study, we aim to compare genomic selection strategies in a simulated population, to investigate their long-term properties with a focus on the preservation of genetic variance, accumulation of genetic gain, and the use and retention of rare alleles and de-novo mutations. These strategies were chosen to represent a range of approaches to balance between genetic improvement and diversity management. To further explore the range of strategies, we developed and tested a constrained allele loss approach that places an explicit constraint on the reduction in frequency of rare favorable alleles. Long-term recurrent selection using each of the five strategies was tested on three traits, with different combinations of additive and non-additive gene actions, simulated following [8].

**Fig. 1:**
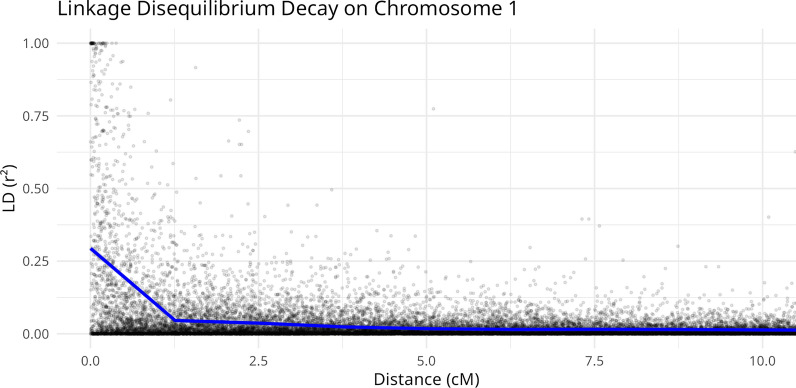
Linkage disequilibrium (measured as $r^2$) against distance (in cM) between pairs of markers in the initial generation of selection (generation 0). The blue line indicates the smoothed trend

**Fig. 2:**
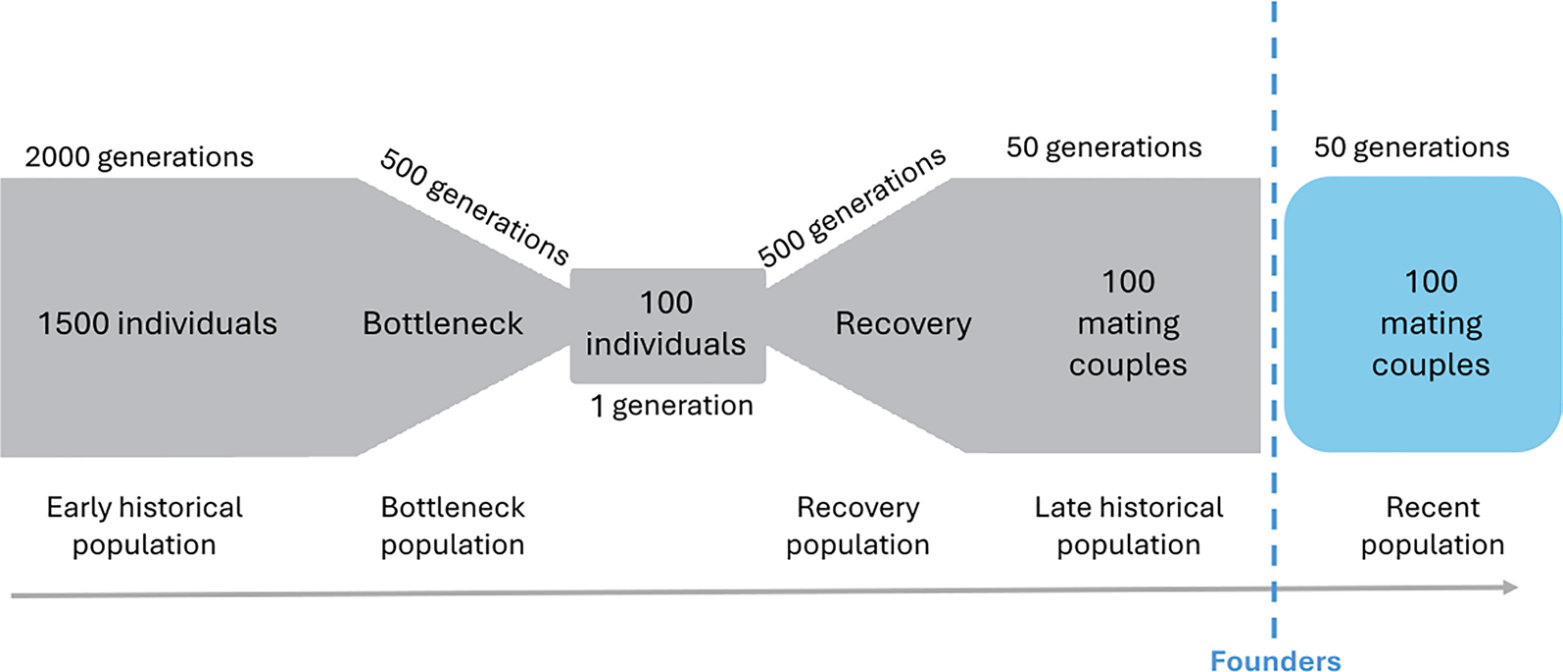
Diagram of the simulation process. The “Recent population” (ie., the last 50 generations) was used to evaluate the different genomic selection strategies

**Fig. 3:**
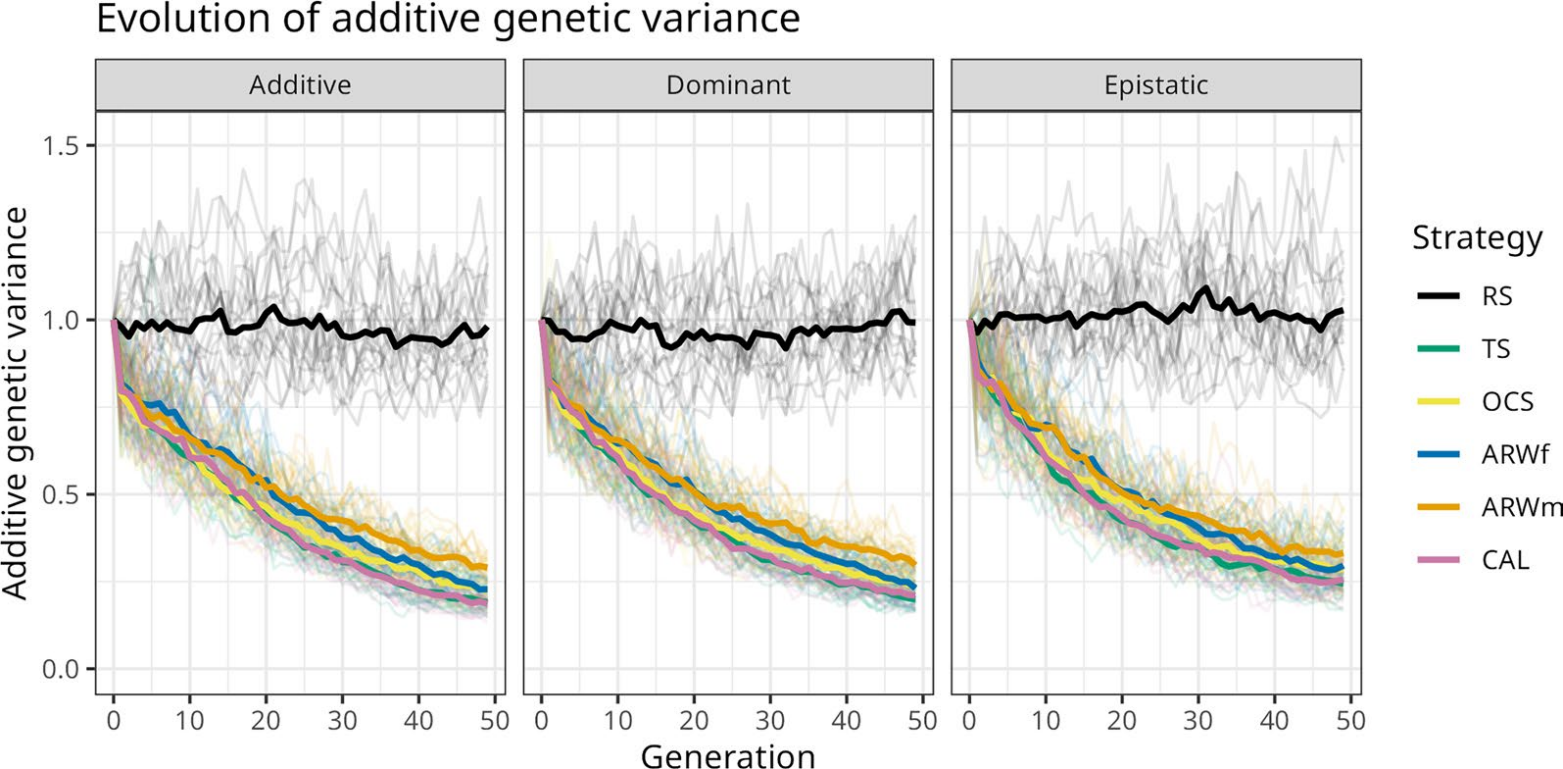
Evolution of additive genetic variance of the population through the generations using five genomic selection strategies. In color the selection strategies: random (RS), truncation selection (TS), optimal contributions (OCS), allele re-weighted with fixed time horizon (ARWf) and with moving time horizon (ARWm) and constrained allele loss (CAL). Light lines are used for the individual simulation replicates, in bold, the averages across replicates

**Fig. 4:**
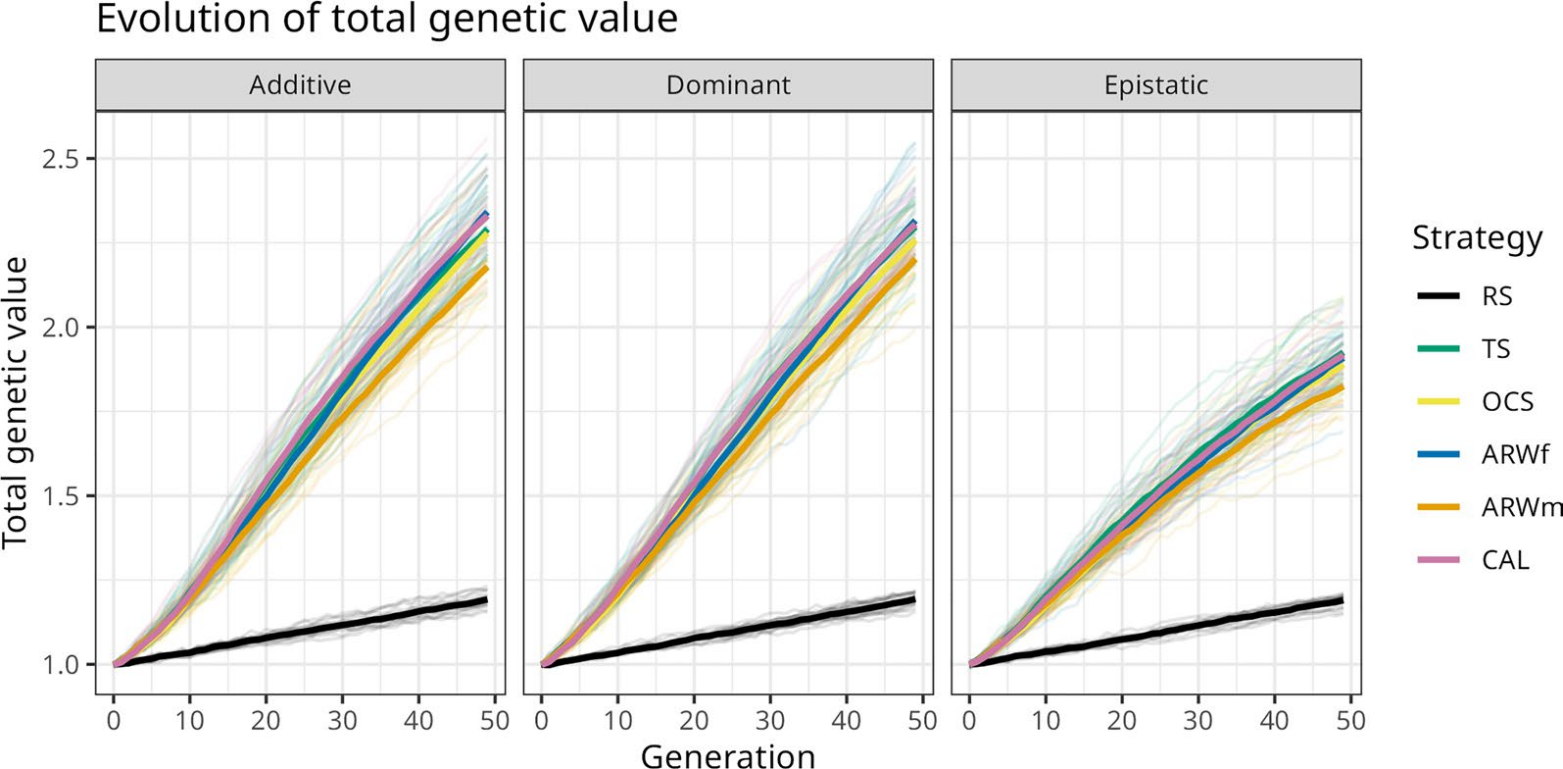
Evolution of the average total genetic value of the population through the generations (expressed in units of genetic standard deviations). In color the selection strategies: random (RS), truncation selection (TS), optimal contributions (OCS), allele re-weighted with fixed time horizon (ARWf) and with moving time horizon (ARWm) and constrained allele loss (CAL). Light lines are used for the individual simulation replicates, in bold, the averages across replicates

**Fig. 5:**
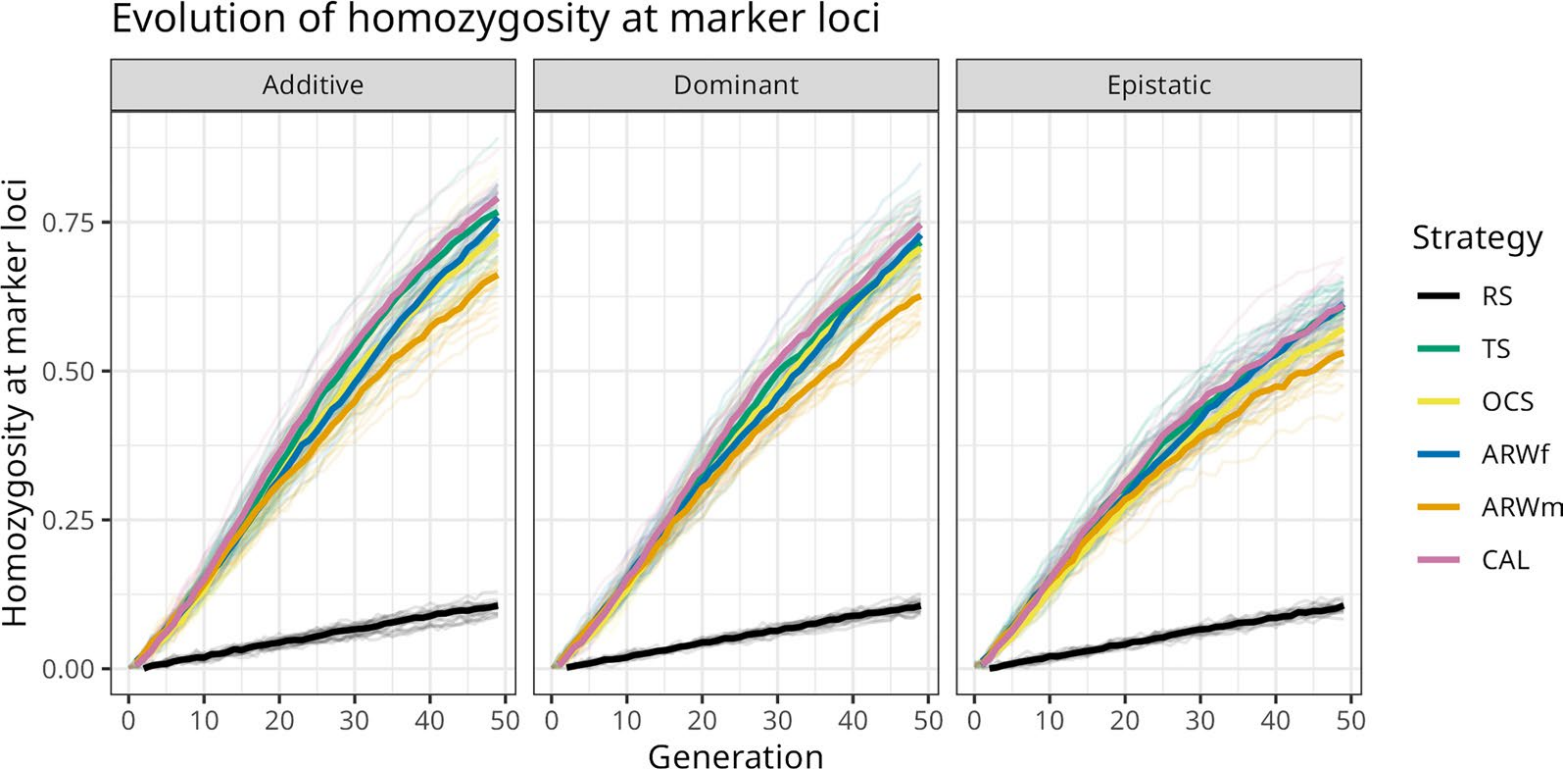
Evolution of homozygosity of the population through the generations using five genomic selection strategies. In color the selection strategies: random (RS), truncation selection (TS), optimal contributions (OCS), allele re-weighted with fixed time horizon (ARWf) and with moving time horizon (ARWm) and constrained allele loss (CAL). Light lines are used for the individual simulation replicates, in bold, the averages across replicates

## Methods

To explore the long-term effects of five genomic selection strategies, we simulated a livestock population with 50 generations of recurrent selection. The simulation followed the setup described in [8]. For the sake of completeness, the details of the simulation are first provided. Second, we describe in detail the five genomic selection strategies.

**Fig. 6:**
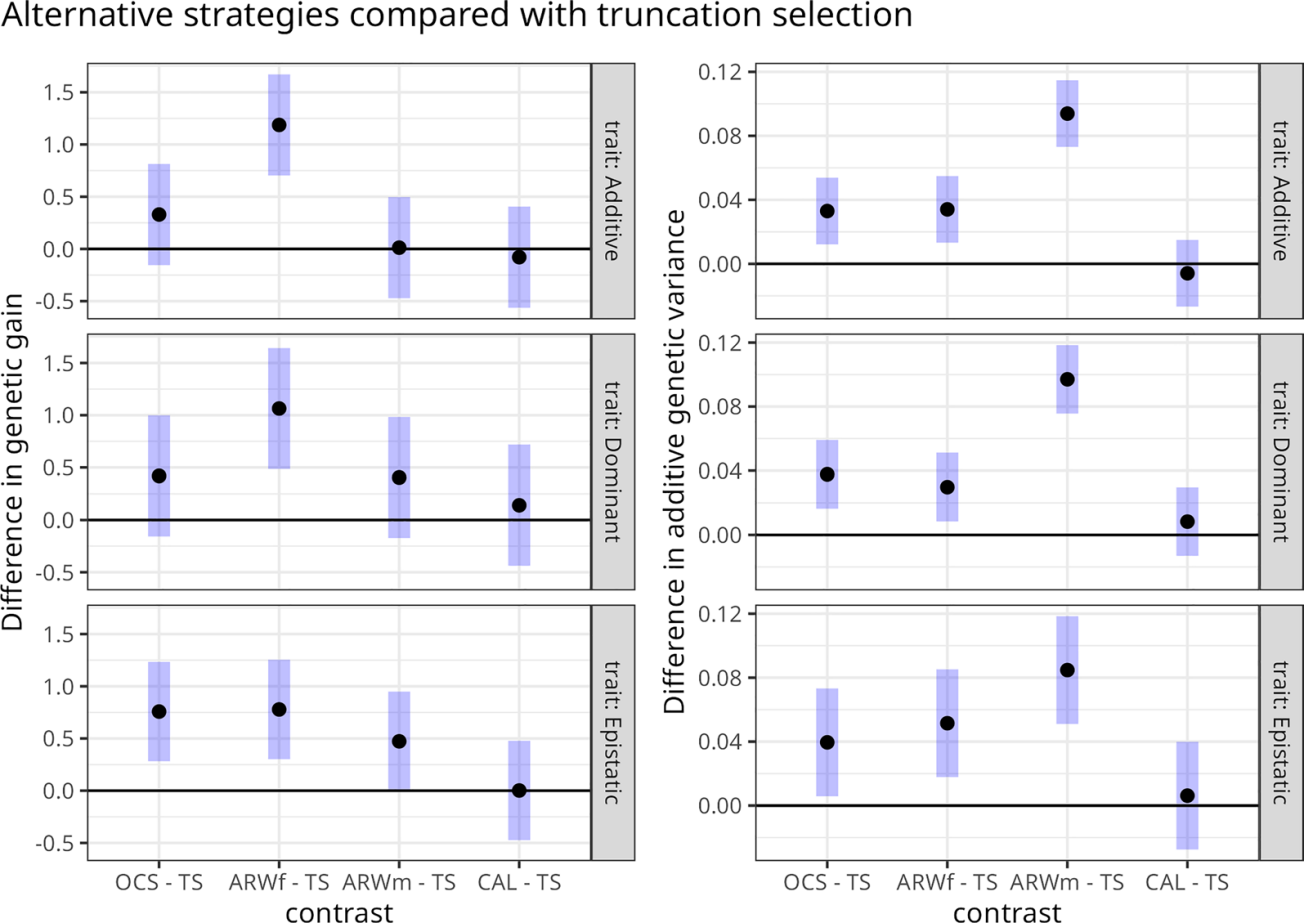
Alternative selection strategies compared with truncation selection in terms of the accumulated genetic gain (left panel) and the additive genetic variance (right panel) after 50 generations of recurrent selection. 95% confidence intervals adjusted for multiple comparisons

**Fig. 7:**
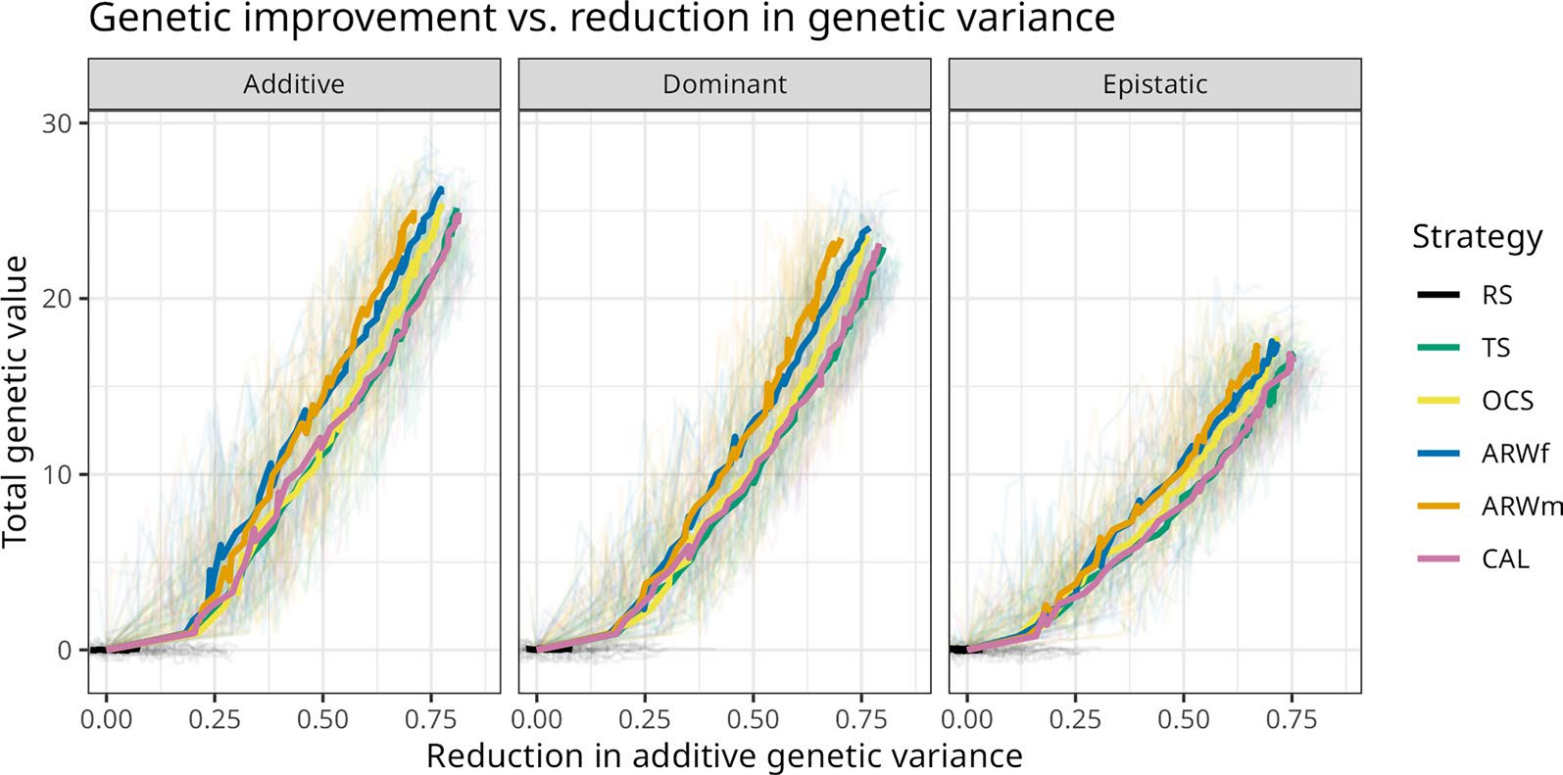
Reduction in additive genetic variance against total genetic value. In color the selection strategies: random (RS), truncation selection (TS), optimal contributions (OCS), allele re-weighted with fixed time horizon (ARWf) and with moving time horizon (ARWm) and constrained allele loss (CAL). Light lines are used for the individual simulation replicates, in bold, the averages across replicates

**Fig. 8:**
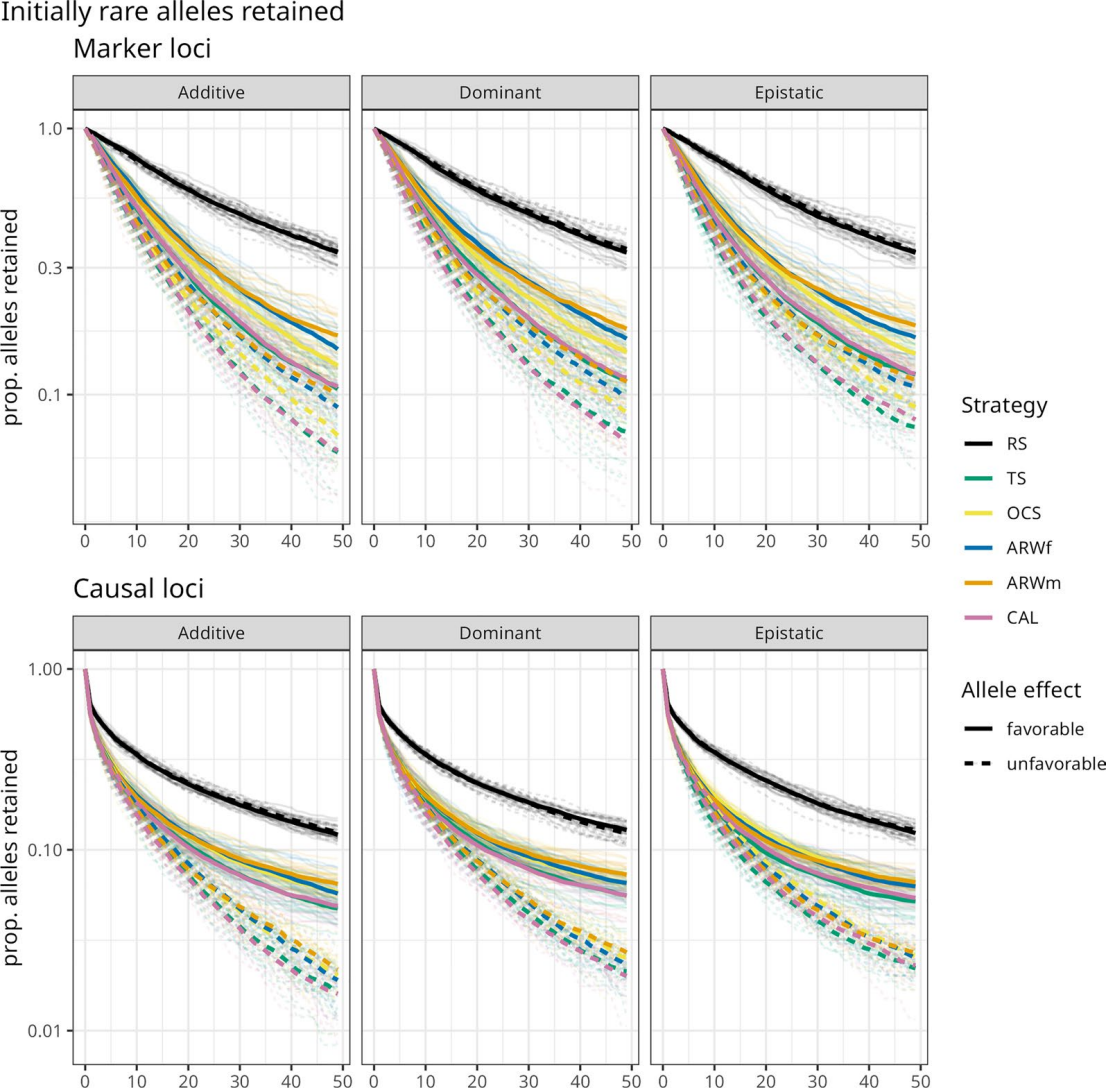
Proportion of initially rare alleles still segregating in the population through the generations, for marker loci (top panel), and causal loci (bottom panel). In color the selection strategies: random (RS), truncation selection (TS), optimal contributions (OCS), allele re-weighted with fixed time horizon (ARWf) and with moving time horizon (ARWm) and constrained allele loss (CAL). Light lines are used for the individual simulation replicates, in bold, the averages across replicates. Standard errors are indicated in gray. Note that the y-axis scale is different between the panels

**Fig. 9:**
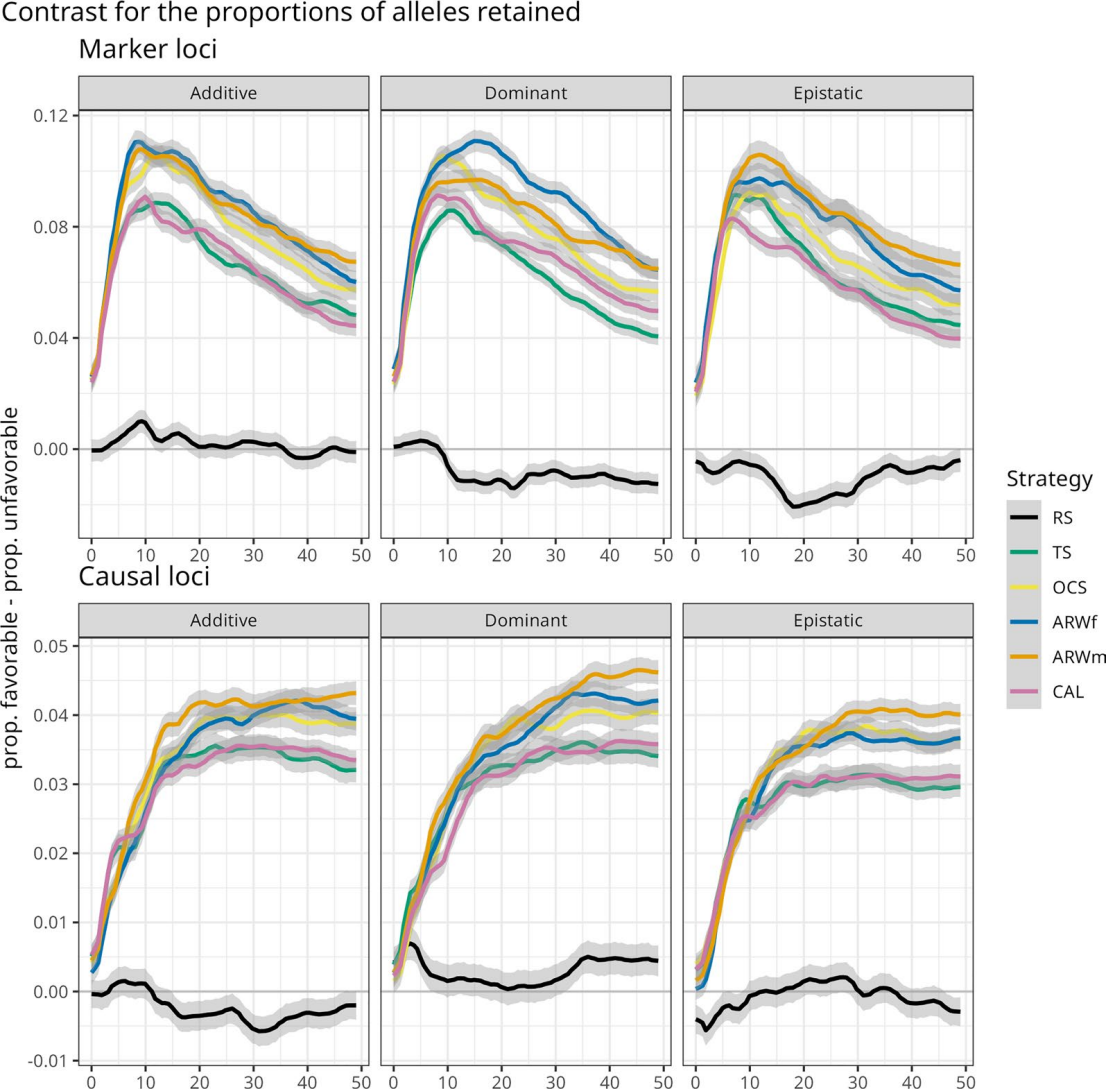
Contrasts for the proportions of initially favorable and unfavorable rare alleles for marker loci (top panel), and causal loci (bottom panel). In color the selection strategies: random (RS), truncation selection (TS), optimal contributions (OCS), allele re-weighted with fixed time horizon (ARWf) and with moving time horizon (ARWm) and constrained allele loss (CAL). Light lines are used for the individual simulation replicates, in bold, the averages across replicates. Standard errors are indicated in gray. Note that the y-axis scale is different between the panels

**Fig. 10:**
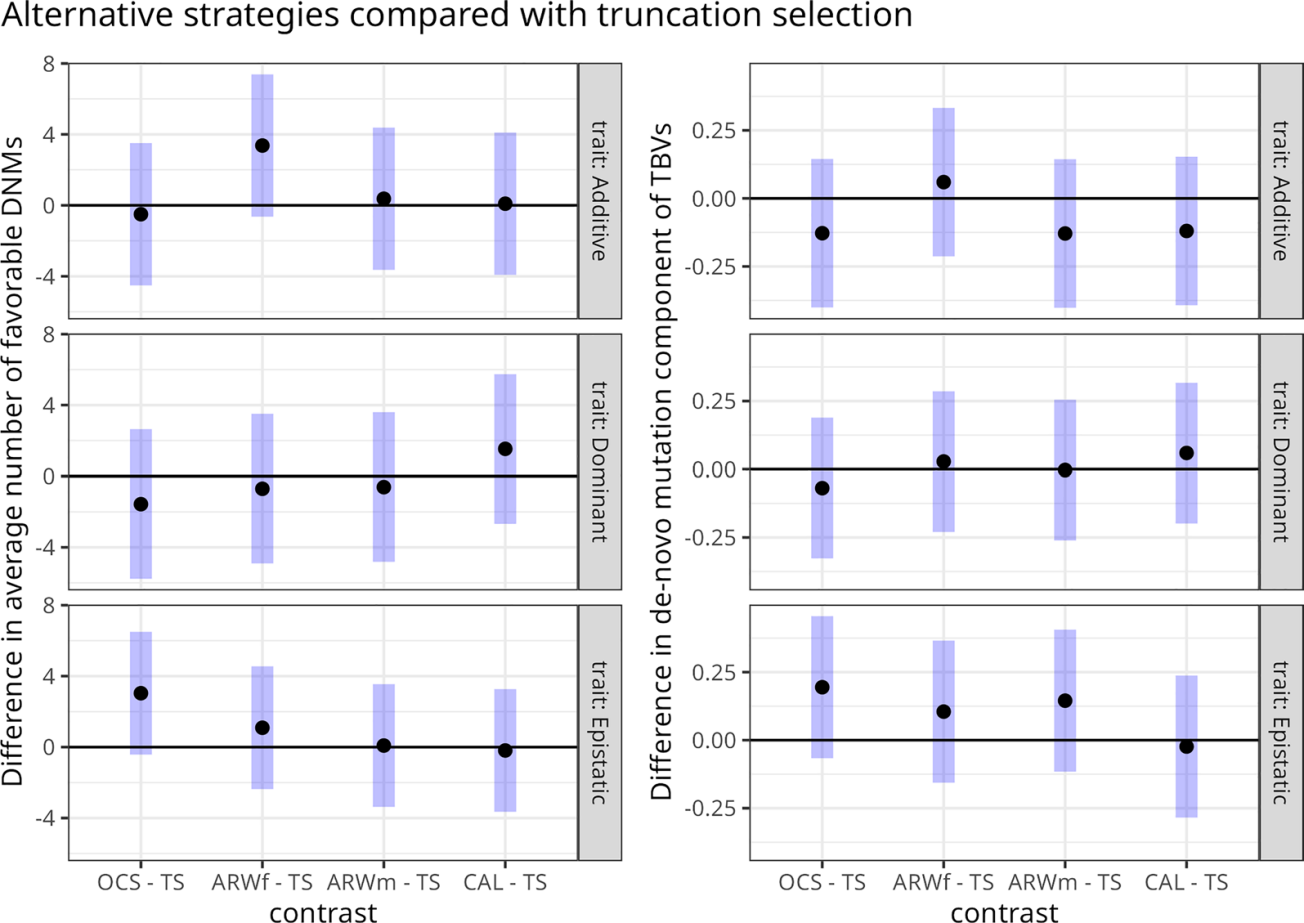
Excess of favorable de-novo mutations (number of favorable mutations minus number of unfavorable mutations accumulated; left panel) and component of the additive genetic value contributed by de-novo mutations (number of favorable mutations minus number of unfavorable mutations accumulated; right panel) after 50 generations of recurrent selection for different selection strategies compared with truncation selection. 95% confidence intervals adjusted for multiple comparisons

### Simulated population

To obtain the founder generation we first generated a historical population using the QMSim software [9]. This historical population consisted initially of 1500 individuals, randomly selected and mated for 2000 generations. This was followed by a linear decrease in population size for 500 generations until a minimum of 100 individuals, to generate a linkage disequilibrium pattern similar to that observed in livestock populations (see Fig 1). This bottleneck was inmediately followed by a linear increase up to 1500 individuals over 500 generations. Then a 100 pairs of mating couples were randomly selected and evolved for a further 50 generations using a custom Fortran program. These last 50 generations of the historical population had litter sizes of 10, with 1:1 sex ratio; in each generation a 100 females and a 100 males were kept and randomly mated. The offsprings from the last historical generation constitute the founder generation for the recent population. In the recent population, the 100 sires and dams were selected according to one of the five selection strategies described below (see section “Selection strategies”). The recent population was evolved for 50 generations, with the same mating scheme as the last 50 generations of the historical population. The numbers of sires, dams, and progeny in a litter were chosen to keep results comparable with [8] and [10]. The simulation was repeated 20 times to obtain a total of 20 replicates. A diagram of the simulation scheme is shown in Fig. 2.

### Marker panel, causal loci and de-novo mutations

The genome was simulated with 10 chromosomes, each chromosome of one Morgan length and with 200,000 randomly spaced bi-allelic loci in the historical population. During the first phase of the simulation, all loci were simulated with a recurrent mutation rate of $5 \times 10^{-5}$ to maintain at most two alleles at a locus. At the end of the expansion (i.e., the beginning of the “late historical population” and fifty generations before the founders of the selected population), 20 thousand marker and two thousand causal loci were chosen from the segregating bi-allelic loci. Firstly, the marker loci were sampled to have an uniform allelic frequency distribution, with a minimum minor allele frequency (MAF) of 0.01. This aimed to mimic the distribution found in commercial SNP marker panels. The resulting density of 20 markers per cM is similar to that of widespread commercial SNP panels used in livestock, such as 50k markers ofent used for cattle [11]. Higher densities have been shown to provide only marginal improvements in the accuracy of genomic selection [12], and panels of lower densities can be cheaply imputed to this densities with imputation [13]. As for the causal loci, these were sampled with equal probability from all remaining segregating loci, replicating the U-shaped allelic frequency distribution arisen in the historical population. Finally, four thousand causal loci with effects on the trait, which were not segregating at the end of the expansion, were randomly chosen to be candidate sites for de-novo mutations throughout the following generations. Although fixed loci carry no observable statistical effect in the absence of genetic variation, the mutations arising at these sites in subsequent generations created new variation at loci that already have functional effects on the trait (as described in the following section). Specifically, these candidate mutation sites were mutated in the following generations with an expected number of 0.6 de-novo mutations for each individual (with the specific number of mutations per individual being Poisson-distributed). The chosen mutation rate resulted in a mutational variance of $\sim 0.001\sigma ^2_{\text {e}}$ for the additive trait, as explained in [8]; this is within the range often observed in real populations [14, 15]. As most de-novo mutations are lost in the first generation, the fixated loci among these four thousand candidate loci were reused throughout the simulation. This procedure was followed to be comparable with previous work [8, 10], and as a compromise between computational efficiency and biological realism.

### Simulated traits

Following [8], we simulated three traits: a trait with only additive effects (“additive”), a trait with additive and dominance effects (“dominant”), and a trait with additive, dominance, and epistatic effects (“epistatic”). For all traits, an allele was selected randomly for each causal locus *i*, and this allele was assigned an additive effect ($a_i$) sampled from a standard normal distribution (i.e., mean 0.0 and standard deviation 1.0). To generate dominance, first a degree of dominance ($dd_i$) was sampled for each locus from a normal distribution with mean 0.2 and standard deviation 0.3. Then the functional dominance effect ($d_i$) was obtained by multiplying this degree with the absolute value of the corresponding additive effect for the same locus ($d_i = dd_i|a_i|$). The positive mean in the degree of dominance distribution results in a bias for overdominance which was observed in both natural and livestock populations [16]. This overdominance also implies a non-zero inbreeding load. Following eq. 14.4 in [17], the inbreeding load $B = 2\sum _j d_j p_j q_j$ was calculated for the dominant trait at the founder generation. Expressed as the percentage decrease in mean phenotype per 10% increase in inbreeding coefficient, relative to the phenotypic standard deviation, the inbreeding load in the simulation ranged from 13.4% to 21.0% across replicates; with a mean of 18.3%. These values are consistent with the range of 4–51%; with a mean of 22%, reported across diverse traits and species by [17]. They exceed the range of -7% to 12%; with a mean of 5%, estimated for livestock production traits by [18], which is not unexpected: empirical livestock estimates reflect populations with long selection histories during which mildly deleterious recessive alleles may have been partially purged, whereas the simulated population here was parameterized from a distribution of dominance effects without such prior purging. Similarly to the dominance effects, functional epistatic effects ($e_{ij}$) were simulated to be proportional to the absolute value of the additive effects of the interacting loci: for loci *i* and *j*, $e_{ij} = \varepsilon _{ij}\sqrt{|a_ia_j|}$, where the degree of epistasis ($\varepsilon _{ij}$) was sampled from a normal distribution of mean 0 and standard deviation 0.45, independently for each of the 9 possible genotypic combinations for the loci *i* and *j*. Only pairwise epistasis was simulated, and the pattern of interactions was modeled after the connectivity pattern observed in yeast between $\sim 6000$ genes, with many loci with few interactions and few loci with many interactions [19]. The functional genetic effects were combined with the genotypes of the individuals to calculate total genetic values. To obtain the simulated phenotypes, the total genetic values were added to residual terms, sampled from a normal distribution with mean zero and a variance equal to 1.5 times the total genetic variance at the start of the selection scheme (generation $t=0$), resulting in an expected broad sense heritability of 0.4 in this initial generation.

### Statistical effects

There is a distinction to be made between genetic effects measured as simple contrasts between phenotypic means of genotypes (called *functional effects*), and those which describe their contribution to different population-level variance components and which aim (at least under simplified assumptions) to be orthogonal (*statistical effects*). This distinction is important because different genetic components can contribute to the same variance component: in particular, the dominance functional effects described above have both an additive and a dominant statistical component; while the epistatic functional components contribute to epistatic, as well as to dominant and additive statistical components [20].

To properly quantify additive genetic variation and breeding values in the presence of dominance and epistasis, we need to decompose functional effects into their statistical components. For this we used the Natural and Orthogonal Interaction (NOIA) procedure [21] and obtained statistical additive effects. These statistical additive effects were used to: 1) calculate true breeding values, 2) calculate additive genetic variation, 3) characterize causal alleles as favorable or unfavorable by the sign of their statistical additive effect, and 4) calculate the component of the true breeding value contributed by de-novo mutations. Alternative approaches for this decomposition exist (e.g., classical least-squares methods), but NOIA provides a systematic framework that explicitly accounts for allele frequency changes and maintains orthogonality between variance components. For readers interested in the conceptual and mathematical foundations underlying the relationship between functional and statistical effects, we refer to [20, 21], which cover complementary aspects of this topic.

### Selection strategies

In this work we compared the long-term performance and consequences of five different genomic selection strategies. The strategies were chosen to represent a variety of approaches to balance between genetic improvement and diversity management. Briefly, these selection strategies are truncation selection (TS), optimal contribution selection (OCS) [4], two versions of allele-reweighted selection (ARW) [5], and a novel constrained allele loss selection (CAL). Finally, a scenario with random selection (RS) was also included as a reference. These five strategies were evaluated to identify the ones which best conserve favorable rare alleles and de-novo mutations in the presence of additive and non-additive gene actions.

Except for RS, all strategies involved the prediction of genomic estimated breeding values (GEBVs) for the selection candidates. These GEBVs were calculated from marker effects (${\boldsymbol{{\hat{\beta }}}}$), predicted with ridge-regression [22] using the genetic and phenotypic information of the previous three generations. The marker regression used a common shared intercept and a '012' allele coding [23]. Variance components were also estimated each generation, by Restricted Maximum Likelihood (REML) [24] using the information from the three previous generations.

#### Random selection

This strategy was included as a reference, to characterize the trends and variation of the statistics measured which are due to drift in the absence of a directional selection. With this goal, the generations were evolved following the same process used for the last 50 generations of the historical population (see section “Simulated population”).

#### Truncation selection

This strategy consists of choosing as sires and dams those candidates with the highest GEBVs to produce the next generation. This aims to maximize expected genetic gain in the short-term, but does not take any consideration of rare alleles in particular, or for diversity management in general. Because marker-regression is equivalent with Genomic Best Linear Unbiased Prediction (G-BLUP) [23], and no phenotypes of the selected candidates were used in the GEBV prediction, this strategy aligns with the scenario “GBLUP_NoOP” explored in [2] and [8].

#### Optimal contributions selection (OCS)

The OCS strategy aims to maximize the average breeding value for the set of selected candidates while satisfying a constraint on their average relatedness *K* [4]. Under random mating, the increase in average relatedness ($\Delta K$) is the expected rate of inbreeding ($\Delta F$) in the next generation, which is inversely related to the effective population size ($N_e = 1/(2\Delta F)$) [17]. We included this approach to explore whether avoiding a strong reduction in effective population size could be sufficient to lower the rate of allele loss due to genetic drift from that observed with TS, even without an explicit focus on the rare alleles.

Under OCS, candidates in each generation were ranked according to their optimal contributions, and the top 100 males and 100 females were selected to become parents of the next generation. To obtain the vector of optimal contributions (${\textbf{c}}$), the following optimization problem was solved for,

1 $$\begin{aligned} \text {maximize:}\,\,g&= {\textbf{c}}'{\textbf{X}}{\boldsymbol{{\hat{\beta }}}} \end{aligned}$$

2 $$\begin{aligned} \text {subject to:}\,\,K_t&\ge \frac{1}{2} {\textbf{c}}'\textbf{Rc} \end{aligned}$$

3 $$\begin{aligned} \textbf{Qc}&= \left[ \frac{1}{2}\,\frac{1}{2}\right] ' \end{aligned}$$

4 $$\begin{aligned} {\textbf{c}}&\ge \textrm{0} \end{aligned}$$

where ${\textbf{X}}$ is the allele count matrix (with ’012’ allele coding) for the selection candidates, $K_t = K_{t-1}+(1-K_{t-1})/(2N_e)$, ${\textbf{Q}}$ is a 2-column matrix which assings candidates into sexes, and ${\textbf{R}}$ stands for the relationship matrix between candidates.

It should be noted that the formulation above represents a relaxation of a more theoretically precise implementation. When all selected individuals contribute equally to the next generation, as in this simulation, the objective function $g = \mathbf {c'X}{\boldsymbol{{\hat{\beta }}}}$ under the classical continuous OCS does not equal the expected genetic gain under the equal-contribution reproduction scheme. A more appropriate formulation would use a dichotomic decision variable ($c_i \in \{0, 1\}$) for each candidate, with the constraint that the sum of selected males and females each equals 100. However, this reformulation results in a mixed-integer quadratic program, which is considerably harder to solve than the continuous relaxation. This can be solved through multiple heuristics, including greedy and stochastic optimization approaches, but they are less standardized than the classical continuous formulation [25]. We therefore used the continuous OCS as a ranking tool: the optimal contributions ${\textbf{c}}$ were computed and the top 100 males and 100 females were selected accordingly. This approximation is expected to yield somewhat sub-optimal parent sets relative to a true discrete OCS; however, the diversity management properties of the strategy relative to truncation selection are not expected to change qualitatively [26].

We used the usual pedigree-based numerator relationship matrix [27], and considered the candidates in the first generation of selection as unrelated. In practice, considering the founders as unrelated could be alleviated by using metafounders [28], but as the focus of the current work was on the long-term trends, this was not explored here. While genomic relationship matrices (GRM) could alternatively be used in OCS, the choice of relationship matrix has important consequences for the optimization objective. When using GRMs with reference allele frequencies of 0.5, OCS maximizes expected heterozygosity rather than minimizing drift [29], which can lead to loss of rare alleles. Moreover, it was demonstrated [30] that pedigree-based OCS (POCS) can realize more genetic gain than genomic OCS (GOCS) at the same rate of true inbreeding, because POCS manages expected genetic drift without restricting selection at QTL, whereas GOCS penalizes allele frequency changes at markers in linkage disequilibrium with QTL. Alternative GRM formulations such as those based on linkage analysis [4] may address these issues but lack established implementation methods. Given these considerations and the well-characterized properties of pedigree-based relationships for controlling drift, we chose to use the pedigree-based numerator relationship matrix for OCS.

Finally, $N_e$ stands for target effective population size, and a value of 50 was used here. For comparison, the theoretical value of $N_e$ for RS is 200, and the median $N_e$ for TS was 57 (based on the pedigree inbreeding rate). The $N_e$ for OCS was chosen to be similar as for TS, but slightly lower, with the consideration that pedigree inbreeding can underestimate genomic inbreeding for a population under genomic selection [4]. This $N_e$ is within the range of values observed in livestock populations [31].

#### Allele re-weighting strategies

The allele re-weighting (ARW) strategy, proposed by [32] and further developed in [5], consists of conducting truncation selection on weighted GEBVs (wGEBVs). This strategy upscales the effects of rare loci in the calculation of GEBVs to trade-off the current short-term improvement against the missed long-term potential that would occur from losing potential favorable rare alleles.

The wGEBVs are computed as in [5]:

5 $$\begin{aligned} \textbf{wGEBV} = {\textbf{X}}{\textbf{W}}{\boldsymbol{\beta }} \end{aligned}$$

where ${\textbf{W}}$ is a diagonal matrix with the following weights for locus *j*,

6 $$\begin{aligned} w_{jj} = \frac{(2p_j)^{a_t-1}}{B(a_t,1)}, \end{aligned}$$

where $p_j$ is the frequency of the allele currently estimated to be favorable for marker *j*, *B* is the beta function, and $a_t$ is a parameter which varies at each generation *t*. The parameter $a_t$ regulates the strength of the up-scaling and is calculated as,

7 $$\begin{aligned} a_t = a_o + t \frac{(1-a_o)}{T} \end{aligned}$$

such that it starts at an initial value of $a_o$ when $t=0$, and approaches a value of 1 when *t* approaches *T*, the number of generations in a predetermined time horizon. When $a_t$ equals 1, the weights are equal to 1 and the selection strategy behaves as the truncation selection on the unweighted GEBVs.

A brief sensitivity analysis for the choice of the scaling constant $a_o$ (values 0.1, 0.2 and 0.5) is provided in Appendix A; the analysis shows that the ARW behaviour is moderately robust to $a_o$ while some tuning may improve the performance of the strategy.

Two variants of this strategy were explored here. For the allele re-weighting strategy with a fixed time-horizon (ARWf), the time horizon spanned the whole number of generations under selection ($T=50$). For the allele re-weighting strategy with a moving time-horizon (ARWm) instead, the time horizon was kept at a constant 5 generations ahead (i.e., fixing *T* to 5 and *t* to 1 in Eq. (7)). For both variants, $a_o$ was set to 0.2 as in [5].

#### Constrained allele loss

This novel strategy was designed to explore the potential of a more direct approach to the conservation of rare alleles. It measures the rarity of the alleles estimated to be favorable, and limits the reduction in frequency of those alleles towards the next generation (the lower the allele frequency, the stronger the constraint in the selection criteria for further reduction in frequency). It is similar to the OCS strategy, where the constraint on the relatedness among selected candidates is replaced with a constraint on the rarity of favorable alleles after selection. Concretely, the new constraint is as follows:

8 $$\begin{aligned} L \ge \mathbf {c'X}{\boldsymbol{\alpha }} \end{aligned}$$

where

9 $$\begin{aligned} \alpha _j = -log(1/n \cdot (1 + (\mathbf {J'X})_j)) [if\,\,\beta _j \ge 0], \end{aligned}$$

${\textbf{J}}$ is an n-length vector of ones, and [*if P*] is an Iverson bracket (which equals 1 if *P* is true, and 0 otherwise). The value of *L* was calculated in each generation as $L = 1.10 \cdot 1/n \cdot (\mathbf {J'X}{\boldsymbol{\alpha }})$. Thus, a 10% increase per generation was allowed for this criterion, which summarizes the overall rarity of those alleles estimated to be favorable.

The measure constrained is an average on the (negative) logarithm of frequencies, related to statistics such as Shannon entropy [33], and rapidly increases in value when the frequency of an allele estimated to be favorable approaches zero. This strategy uses logarithms of allelic frequencies as also done in the second optimal selection strategy "OS-II" in [34]. In that study, the rarity measure was applied directly as a penalty on the selection criterion and was used on a reduced number of purely additive QTLs, which requires an intermediate QTL identification step; here the measure is used on the marker loci and the criterion is implemented as a constraint on the selection candidates, as in OCS.

### Statistical summaries

For each simulation scenario, several variables were extracted from each generation, and summarized by their 10% trimmed mean across the 20 replicates. The trim was used to reduce the impact of outliers, specially for the “epistatic” trait and when measuring empirical variances. We tracked the additive genetic variance in each generation, the average total genetic value, and the contribution to the genetic value from the de-novo mutations. We also tracked the average homozygosity at marker loci as a broad indicator of genomic diversity. It should be noted that marker loci are not strictly neutral, as they were sampled to achieve a uniform allele frequency distribution and their estimated effects are used directly in the selection process; the homozygosity at marker loci should therefore be interpreted as a measure of genomic diversity associated with the selected trait rather than of neutral genetic variation.

In addition, the average number of de-novo mutations, of retained rare alleles in marker loci, and of retained rare alleles in causal loci were also counted each generation. These alleles were classified as ‘favorable’ and ‘unfavorable’ according to the sign of their corresponding true statistical additive effect for the de-novo mutations and other causal loci, and by the estimated effect for the marker loci. Alleles were considered as ‘rare’ when their frequency was below 0.01 in the first generation of selection. The classifications into favorable, unfavorable and/or rare alleles were determined based on the effects and frequencies observed in the first generation of selection.

The additive genetic variance for each generation was calculated as the variance of true breeding values (TBVs) for the individuals in that generation. In turn, the TBVs were obtained by adding the statistical additive effects described in the section “Statistical effects”. The average total genetic value was obtained from the sum of all functional effects described in the section “Simulated traits”. Finally, the contribution of de-novo mutations to the genetic value was taken from the TBVs considering only those loci with de-novo mutations.

To compare the results across the different selection strategies, the additive genetic variance obtained with TS was contrasted with each alternative genomic selection strategies (with the exception of RS), within each trait. These contrasts were estimated using a generalized least square model with trait, strategy and interaction effects, a block effect per simulation replicate, and accommodating for an heterogeneous residual variance by trait. Confidence intervals and hypothesis tests were conducted for these contrasts and corrected for the multiple comparisons.

### Software

The simulation study was conducted with a variety of software for the different tasks. To create the historical populations, the software QMSim was used [9], in combination with scripts from [8] in Fortran [35]. The genomic model to estimate allele substitution effects was fitted using the R package ‘rrBLUP’ [36]. The optimization problems for the OCS and CAL strategies were solved using COSMO [37] through the Convex.jl interface [38]. The remaining simulation steps were conducted using Fortran code from [8], combined with custom scripts in Julia [39] to accommodate for the selection strategies. The results of the simulation were analyzed, summarized and visualized using the R programming language [40] and packages ‘emmeans’ [41], ‘dplyr’ [42] and ‘ggplot2’ [43]. All scripts are available upon request to the authors.

## Results

The performance of the five genomic selection strategies was evaluated over 50 generations of selection across three simulated traits with different additive and non-additive components. We present the results in four main categories: first, the maintenance of additive genetic variance across generations; second, the accumulated genetic gain over time; third, the efficiency of converting genetic variance into genetic gain; and finally, the conservation of rare alleles and the accumulation of de-novo mutations.

Despite being specifically designed to preserve rare alleles during selection, the CAL strategy did not demonstrate performance distinct from truncation selection (TS) in our simulations. This happened across all the metrics examined, including rare favorable allele retention and the use of de-novo mutations. This lack of differentiation between CAL and TS was consistent across the three traits, suggesting that the constraint implemented in CAL was insufficient to affect the long-term properties of the selection strategy. Given this, the results sections will primarily focus on the contrasts between TS and the strategies that did show distinctive behavior (OCS, ARWf, and ARWm).

The trajectory for the additive genetic variance was similar for all three traits considered. It was approximately constant for random selection and decreased when genomic selection was used (Fig. 3). As expected, the largest drop in additive genetic variance was observed in the first generations of selection, by approximately 20% in the first generation of selection. This initial drop was observed with all non-neutral selection strategies, was of similar magnitude across them, and is mainly attributed to the “Bulmer effect” [44]. In the long-term, OCS and both ARW strategies maintained higher additive genetic variance than truncation selection (Fig. 6, right). Among the ARW strategies, the ARWm strategy conserved a slightly higher amount of additive genetic variance than the ARWf strategy, but this difference was less pronounced for the trait with epistatic gene actions (see Appendix A for a sensitivity analysis of the ARW scaling constant $a_o$).

Across generations, the average total genetic value of the population remained constant for random selection and increased with all the genomic selection strategies (Fig. 4). The TS strategy showed the highest genetic gain in the first generations followed by an earlier saturation response. Consequently, it was surpassed by the ARWf strategy for all traits; by generations 24 for the additive, 20 for the dominant, and 23 for the epistatic trait. In the long-term, AWRf showed higher accumulated genetic gain than TS for all traits, while OCS and ARWm also surpassed TS for the epistatic trait (Fig. 6, left). OCS surpassed TS on average by generation 10 for the epistatic trait; while ARWm did it by generation 28. Homozigosity levels at the marker loci followed patterns very similar to those observed for the genetic gain (see Fig. 5).

Regarding the relation between genetic variance and genetic gain, we observe an initial reduction in variance which is attributed to the Bulmer effect and was similar for all strategies. After this loss, we observe that more genetic gain is obtained for each unit of additive genetic variation lost for both ARW strategies and across all traits, when compared to truncation selection (Fig. 7). This advantageous “conversion rate” of variation into genetic gain was maintained in all later generations for the ARWm strategy, while the ARWf strategy converged to the rate observed for TS on the last generations (manifested by the lines becoming parallel).

Across all traits, we observed a higher loss of initially rare marker alleles for scenarios with genomic selection than for RS (Fig. 8, top). The loss was more pronounced for those alleles estimated to be unfavorable than for those estimated to be favorable, and for TS than for the ARW strategies. Similar patterns were observed for initially rare alleles in causal loci (Fig. 9, top), with the difference between TS and ARW strategies being smaller but consistent. We observed an overall excess of retained favorable alleles across all strategies and traits (Fig. 8, bottom), with ARW retaining a greater excess of favorable alleles than TS. A similar pattern, albeit less pronounced, was observed for the causal loci (Fig. 9, bottom).

**Fig. 11:**
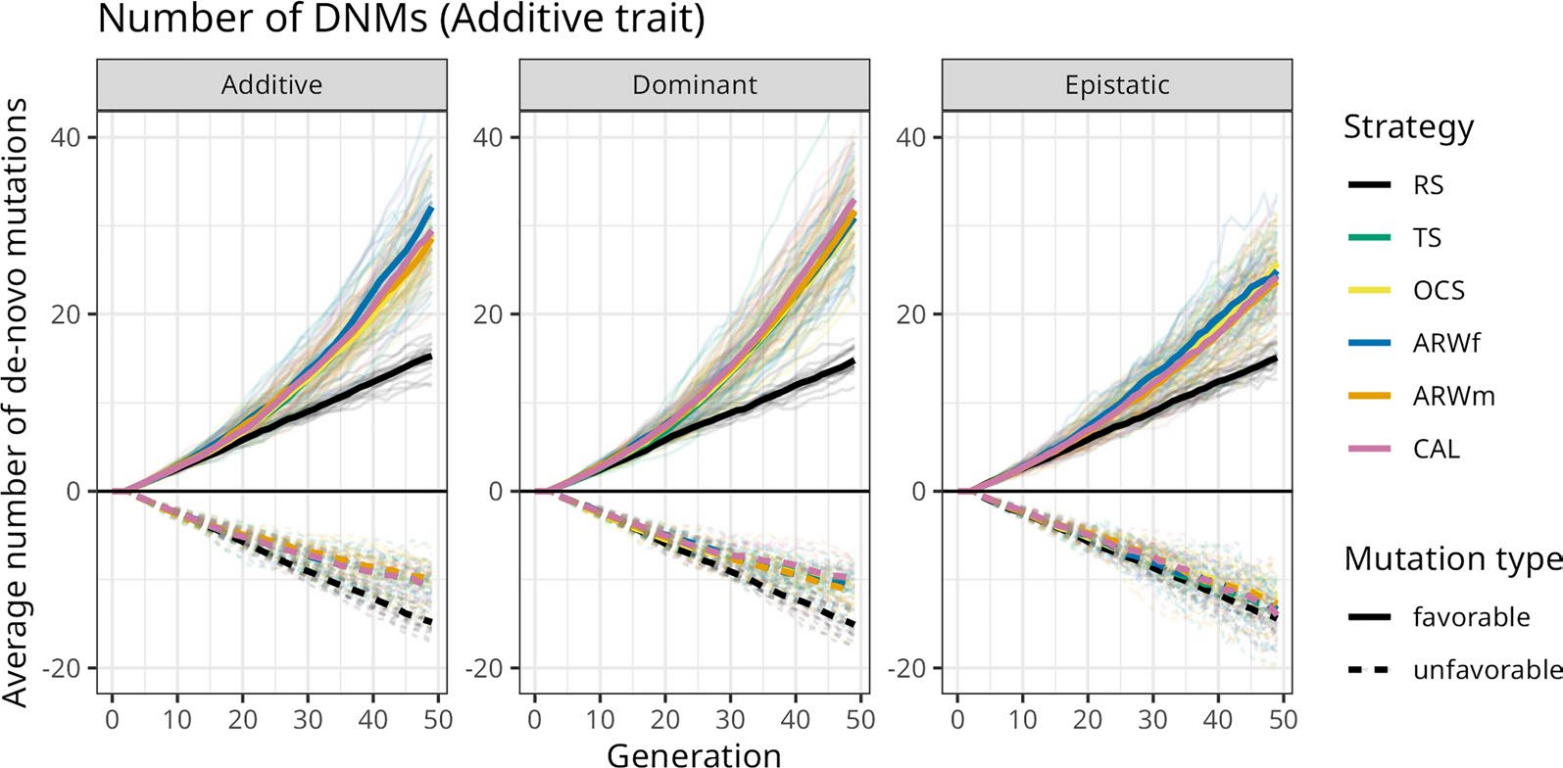
Average number of de-novo mutations per individual accumulated through the generations, both favorable and unfavorable. In color the selection strategies: random (RS), truncation selection (TS), optimal contributions (OCS), allele re-weighted with fixed time horizon (ARWf) and with moving time horizon (ARWm) and constrained allele loss (CAL). Light lines are used for the individual simulation replicates, in bold, the averages across replicates

**Fig. 12:**
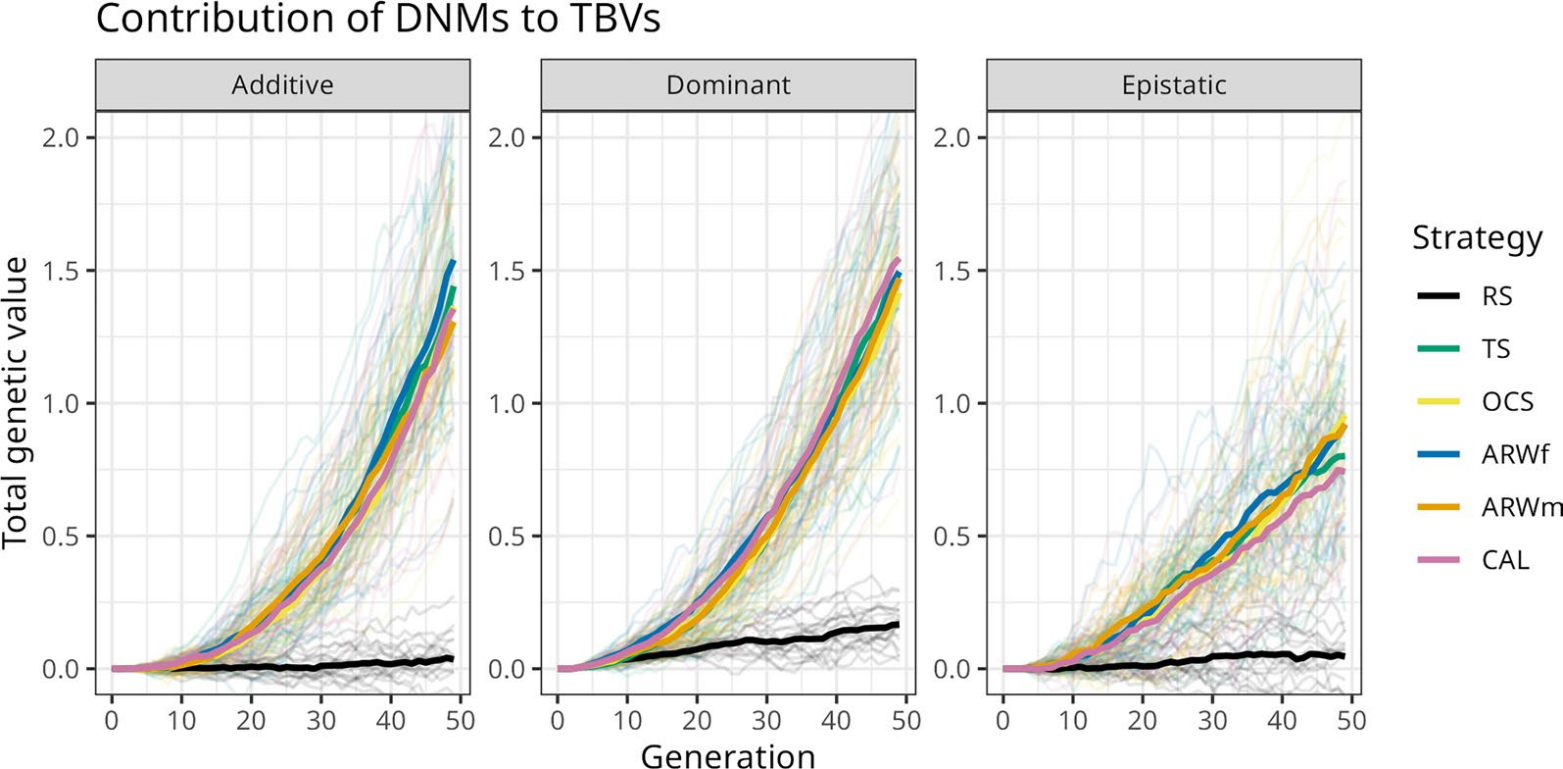
Evolution of the component of the additive genetic value contributed by de-novo mutations accumulated since the base population. In color the selection strategies: random (RS), truncation selection (TS), optimal contributions (OCS), allele re-weighted with fixed time horizon (ARWf) and with moving time horizon (ARWm) and constrained allele loss (CAL). Light lines are used for the individual simulation replicates, in bold, the averages across replicates

With regard to de-novo mutations, all genomic selection strategies behaved similarly, within the stochasticity observed between the simulation replicates (Fig. 10). They increased the frequency of those mutations with positive effects when compared with random selection for all traits (Fig. 11, solid line). For the additive and dominant traits, all genomic strategies accumulated mutations with unfavorable effects at a lower rate than random selection; while for the epistatic trait, the accumulation rate was more comparable (Fig. 11, dashed line). The accumulated contribution of de-novo mutations to the average true breeding value was also equivalent for all genomic selection strategies and surpassed with one genetic standard deviation the results from RS for the additive and dominant traits, while the observed superiority was lower for epistatic trait (Fig. 12).

In summary, we observed distinct patterns between strategies that prioritize short-term gain (TS) versus those that balance gain with diversity maintenance (OCS, ARWf, ARWm), with the exception of CAL, a strategy developed to focus on rare allele preservation which behaved similarly to TS. These patterns varied somewhat across the three traits, indicating how genetic architecture influences the long-term outcomes of different selection approaches.

## Discussion

The aim of this study was to compare different genomic selection strategies in terms of their long-term impacts on accumulated genetic gain and the maintaining of genetic variation. Moreover, we explored the use that these strategies make of de-novo mutations in particular, and of rare alleles in general. We compared five strategies against each other, that is truncation selection (TS), optimal contribution selection (OCS), allele re-weighting strategies (ARW), and a novel constrained allele loss strategy (CAL). These strategies were compared across traits with purely additive and combinations of additive and non-additive genetic effects [8].

Across all traits, we observed that the ARW strategy with a fixed time horizon (ARWf) [5] obtained a higher genetic gain in the long-term while maintaining the same amount of additive genetic variance than TS. Conversely, the modified version of this ARW strategy with a moving time horizon (ARWm) maintained a higher proportion of the additive genetic variance than TS while achieving the same long-term genetic gain for the fully additive and dominant trait; and also surpassing the genetic gain for the trait with epistasis. More generally, fine-tuning the strength of the reweigthing through the parameter $a_o$ could yield improvements depending on specific breeding goals.

This study focused exclusively on the additive genetic variation which impacts the traits under selection. Nevertheless, there is also interest in maintaining genomic diversity beyond what directly impacts the trait under selection, including variation that may affect the trait in the future or impact other traits of interest. This is the focus of diversity management, which aims for the conservation of variation which does not presently impact on the genetic variance of the selected traits [25]. Related to this, we observed an advantage of OCS relative to TS for the long-term genetic gain for the epistatic trait and not for the additive trait. This pattern is consistent with new additive genetic variation in later generations being generated from non-additive genetic variation conserved from the first generations via changes in the allelic frequency of loci with non-additive effects, and we expect to observe this to a higher extent for the epistatic trait [7], and for the OCS strategy more than other strategies [25].

Another limitation of this study is that we did not consider fitness as an explicit trait under selection. In real populations, it is expected that many rare alleles are deleterious and under negative selection. Thus, some of the rare alleles retained by the strategies focused on rare alleles could be deleterious, which would have a negative impact on fitness-related traits. To mitigate this concern, the selected trait could represent a selection index that includes fitness-related traits. Additionally, other traits can be monitored alongside selection for the primary trait to ensure a balanced approach. This concern is particularly relevant to single-trait selection, underscoring the importance of multi-trait selection indices that account for pleiotropic effects.

Three of the strategies considered in this study directly emphasize rare alleles in the selection process. Indeed, the allele re-weighting strategies (ARWf and ARWm) and the constrained allele loss strategy (CAL) all aim to increase the selection pressure on rare alleles, and to maintain them in the population. We confirmed that ARWf and ARWm retain a higher proportion of initially rare alleles than TS, not only in marker loci but also in the causal loci, which are not directly observed by the selection process. For these causal loci, the ARW strategies also retain a higher proportion of initially favorable rare alleles, when compared to the proportion of initially unfavorable alleles retained. The CAL strategy, on the other hand, showed a similar performance to TS in terms of the conservation of rare alleles. The observed reduction in loss rate for the ARW strategies cannot be solely attributed to an increased effective population size, as the effect observed is asymmetrical on favorable and unfavorable alleles. It is plausible that markers with favorable rare alleles are linked to causal loci with similarly favorable rare alleles, though linkage disequilibrium weakens when both loci have rare alleles [45]. Thus, the precise mechanisms by which upweighting rare marker alleles affects the loss rate of rare alleles at causal loci require further investigation. Nonetheless, the study’s findings offer promising implications for utilizing the ARW strategies as in [5, 32], and suggests that a revised implementation of the CAL strategy would be needed if it were to achieve the goal of reducing favorable allele loss.

On the other hand, even though the ARW strategies and CAL all emphasize rare alleles, we observed no difference with respect to TS in the accumulation of favorable de-novo mutations. Neither did we observed an impact on the purging of unfavorable mutations. Of note, the purging of unfavorable mutations was particularly ineffective in the case of the epistatic trait, regardless of the selection strategy considered. This highlights limitations of genomic selection in removing rare, unfavorable alleles; specially without own records for the selection candidates [2, 10]. The limitations can be even greater if unfavourable alleles are recessive, as has long been predicted theoretically [46].

Overall, we see that ARW strategies allow for an increased long-term accumulated genetic gain and a better conservation of genetic variance, when compared with TS on the genomic estimated breeding values. This is remarkable, as the ARW strategies explored were applied on markers instead of causal loci, and the advantages were observed even with non-additive traits where allele substitution effects can change across generations. OCS also showed an advantage over TS in terms of the conservation of genetic variance, and for the epistatic trait also resulted in higher long-term genetic gain. Finally, the CAL strategy showed a similar performance than TS under the metrics considered. While it is possible that a more refined implementation could improve its performance, the results suggest that a lighter focus on the rarest alleles (as that employed by the ARW strategies) should be recommended. More generally, this work tested only a few selection strategies from the wide range of possibilities, and even then used one or a few values of the parameters that regulate these strategies, so it is possible that other strategies and other variations of those presented here could perform better. Still, it is important to further develop strategies that better maintain genetic variation, both in general and specifically in the form of rare alleles, in order to ensure the long-term sustainability of breeding programs which make use of genomic selection.

## Conclusions

In conclusion, this study observed that modified genomic selection criteria, particularly allele re-weighting strategies, may offer advantages over truncation selection by increasing long-term genetic gain and conserving genetic variance. These re-weighting strategies effectively retain a higher proportion of initially rare alleles, contributing to a more sustainable genetic improvement process. Optimal contribution selection also proved beneficial, for the trait with epistatic effects, by preserving genetic diversity and generating higher long-term gains. Conversely, the novel constrained allele loss strategy showed comparable results to truncation selection, indicating a need to revise the criteria if it is to be further explored. Besides the advantages observed in the metrics above, none of the strategies significantly impacted the accumulation of favorable de-novo mutations or the purging of unfavorable ones, underscoring the challenges in managing rare, unfavorable alleles without own records in genomic selection. In general, this study shows the potential of balanced approaches, which by incorporating elements of re-weighting strategies and/or optimal contribution methods, can enhance the sustainability and long-term goals of breeding programs.

## Figures and Tables

**Fig. 13:**
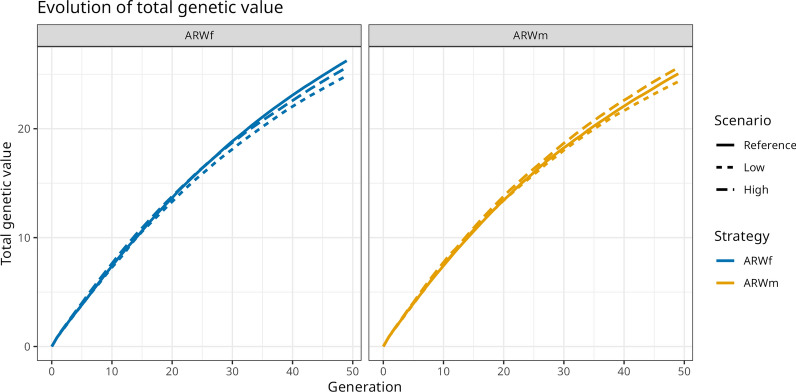
Genetic gain across 50 generations of selection for ARW strategies under three values of the scaling constant. Scenarios (Low, Reference and High) in line type, and selection strategy (ARWf, ARWm) in color. Each scenario was run for 20 replicates (average shown)

**Fig. 14:**
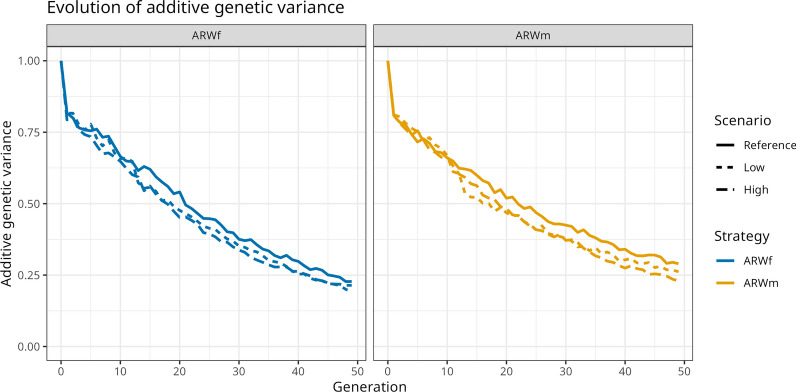
Additive genetic variance across 50 generations of selection for ARW strategies under three values of the scaling constant. Scenarios (Low, Reference and High) in line type, and selection strategy (ARWf, ARWm) in color. Each scenario was run for 20 replicates (average shown)

**Fig. 15:**
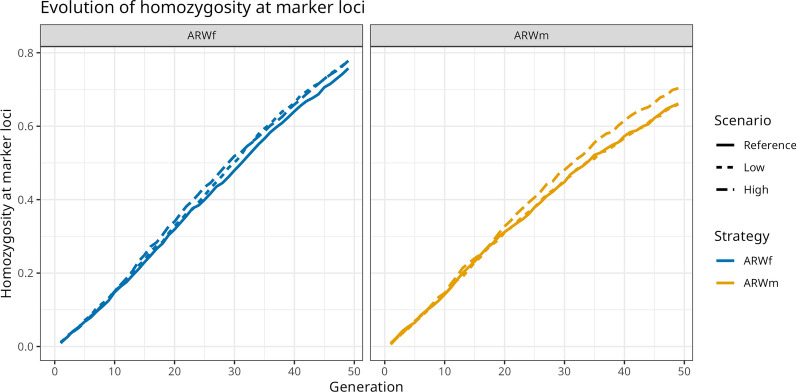
SNP homozygosity across 50 generations of selection for ARW strategies under three values of the scaling constant. Scenarios (Low, Reference and High) in line type, and selection strategy (ARWf, ARWm) in color. Each scenario was run for 20 replicates (average shown)

## Data Availability

All data used in this study was generated with the software specified in the software section. The simulation code is available upon request to the authors.

## Appendix

### Sensitivity analysis for $a_o$ in ARW strategies

This appendix contains a sensitivity analysis of the performance of the ARW strategies [5] to different values of the scaling constant $a_o$.

For the sensitivity runs we re-ran the reference simulation scenario for the additive trait (20 replicates per scenario) while changing only the ARW scaling constant $a_o \in \{0.1, 0.2, 0.5\}$. All other model settings (marker set, genetic architecture, selection scheme and random seeds per replicate) were kept identical to the main experiments so the results are directly comparable.

In addition to the value of $a_o = 0.2$ (“Reference”), we evaluated values of $a_o = 0.1$ (“Low”) and $a_o = 0.5$ (“High”). Figures , and show the results for genetic gain, additive genetic variance and SNP homozygosity across 50 generations of selection, respectively.

As we can see, the different values of $a_o$ produce similar trends across generations, with the ARW strategies not being extremely sensitive to the exact choice of $a_o$ within the range explored. Nevertheless, variations in performance metrics can be observed, even to a similar extent as those observed between different selection strategies. These patterns indicate moderate robustness to $a_o$ but also justify modest parameter tuning for program-specific goals. Notably, the behaviour of the metrics for these strategies are not monotonic with respect to $a_o$ for all metrics. Future research could further explore these behaviours across a wider range of $a_o$ values, or even adaptive strategies for setting $a_o$.
